# Supplementary material for: Conditional prediction of consecutive tumor evolution using cancer progression models: What genotype comes next?
Source: PLoS Comput Biol. 2021 Dec 21;17(12):e1009055. doi: 10.1371/journal.pcbi.1009055 (PMC8730404; doi:10.1371/journal.pcbi.1009055)
Supplement: Attachment — Submitted filename: responses-reviewers.pdf [file pcbi.1009055.s002.pdf]

# Response to reviewers of “Conditional prediction of consecutive tumor evolution using cancer progression models: What genotype comes next?”

Juan Diaz-Colunga, Ramon Diaz-Uriarte

20 October 2021

Dear Reviewers and Editor,

Thank you for giving us the opportunity to submit a revised version of our manuscript PCOMPBIOL-D-21-00878 “Conditional prediction of consecutive tumor evolution using cancer progression models: What genotype comes next?”. We would like to thank you for your time and comments, which have helped us to significantly improve the manuscript.

Reviewer 1 points that “the paper (...) examines important questions for the tumor progression modelling community (...), the contribution is novel and relevant”. Reviewer 2 notes that “this study on its own is interesting and complements the author’s recent lines of research”. Both reviewers, however, express some concerns regarding the readability of the manuscript as well as the biological significance and overall contextualization of our work. Following their suggestions, we have made major changes in the organization of the text and expanded on both the motivation of our question and the forward-looking perspectives of our results. We firmly believe that the manuscript has substantially improved with these changes and is now easier to read and better motivated and discussed.

Below we provide detailed responses to **all** of the reviewers’ comments. Reviewers’ comments are *italicized and shown in green* and changes in the manuscript and Supporting Information are indicated in red (with removed text indicated in ~~stricken through pink~~).

Sincerely,

The authors

## Reviewer #1

*The paper analyses how well Cancer Progression Models (CPM) that are inferred from cross-sectional data can predict short-term progression for a given tumor genotype. To this end, the authors generate cross-sectional data from evolutionary simulations under varying conditions and provide these to five CPMs (CBN, MHN, OT, CAPRI, CAPRESE).*

*In particular, they allow for two important conditions that none of these CPMs can account for:*

*- violation of strong selection and weak mutation (SSWM) such that multiple clones can coexist at a time, whereas CPMs assume that clonal sweeps quickly take over the entire tumor.*

*- fitness landscapes with local maxima, whereas CPMs assume that the completely mutated genotype is always reached as time goes to infinity.*

*Under these conditions, the long-term predictions by CPMs for entire progression trajectories were already found to be poor in previous work. In this paper, the authors focus instead on short-term predictions for a given genotype, which is arguably more clinically relevant. They take great care in formulating this question rigorously as "Given that a genotype with n mutations has been observed, what genotype with n+1 mutations is next in the path of tumor progression (Line of Descent)?" which is well-defined whether SSWM does hold or not. This allows for an extensive comparison of predictive performance for different CPMs under varying simulation conditions.*

### GENERAL COMMENTS

*The paper is generally well written and examines important questions for the tumor progression modelling community, bridging the gap between abstract cross-sectional models and more fine-grained evolutionary models. Although expanding upon similar previous work, the contribution is novel and relevant. The analysis of predictive performance and how it depends on various conditions is extensive and well done. However, is not clear whether these simulations justify strong conclusions for real biological applications.*

We thank the rewiever for their comments. We agree that strong conclusions cannot be drawn regarding real cancer data sets based on our analyis of simulations alone. Part of our results are limited in that simulations may not accurately capture every aspect of cancer progression in a biological context –this, in fact, needs to be generically considered in any work involving models and simulated data. For that reason, our conclusions are toned-down and careful with respect to this limitation. In addition, we have made this more clear in the Discussion section by acknowledging that further work is required to map the transition probabilities (in the sense they are defined in our work) to the evolution of real tumors (see also comment #2 by reviewer #2). We now also refer to this limitation more explicitly in the Discussion, in a paragraph that also incorporates (reworded) sentences from the former version of the Discussion:

> "As is common to simulation work, our results might be of limited value for the analysis of experimental data if our simulations fail to capture key aspects of tumor progression relevant for the problem addressed. Moreover, the analyses discussed above make use of full information from the simulated data that is of course not available in the analysis of real experimental data sets. In the absence of that information, for the analysis of the 25 cancer data sets, and to try to identify genotypes that show potential for accurate consecutive prediction, we used fairly stringent conditions of similarity of predictions within the CE methods (between CBN and MHN), or within the TD methods (between CBN_td and MHN_td). As seen in Fig. 4, for 11 of the data sets, more than 10% of the genotypes meet these stringent conditions: which among the predictions that fulfill those similarity conditions should we trust? The difficulty of this choice is even more dramatic when the same genotype simultaneosuly shows large similarity within TD methods, large similarity within CE methods, and large dissimilarity between the CE and TD methods, as indicated by the gray points in Fig. 4. In these cases, predictions between families of methods are very different and yet method (or method family) choice would require fitness landscape-specific, evolutionary model-specific and genotype-specific information."

### SPECIFIC COMMENTS, MAJOR:

1. *Since CBN, MCCBN and MHN cannot account for fitness landscapes with local maxima, the authors additionally introduce time-discretized variants of these models from their uniformized transition rate*

*matrices. It is not immediately clear to me why line 206 "we will interpret the diagonal entries of the time-discretized transition matrix as a lower bound on the probability that the given genotypes behave as local maxima" is justified biologically.*

The biological justification (for using the diagonal entries of the time-discretized transition matrix) is to try to capture the idea of "remaining in the same genotype" for methods that do not allow for local maxima. We have tried to clarify this further in the manuscript by toning down the initial sentence and expanding on its meaning:

> "To try to overcome this limitation without making specific additional assumptions about the sampling process, and for MHN, CBN, and MCCBN, (...). We will interpret the diagonal entries of the time-discretized transition matrix as a lower bound on the probability that the ~~given~~observed genotypes behave as local maxima, i.e. that no genotype conforms to our criteria of consecutiveness with respect to the observed one and therefore there is a non-zero probability to stay in it."

As we explain also below, these values are not arbitrary, and we say so in the ms. now:

> "Importantly, the probablities that we are interpreting as corresponding to "staying in the observed genotype" are not arbitrary, but based on the underlying statistical models of the time-discretized methods. These may differ significantly in their predictions for staying in the observed genotype, which can give rise to differences in performance."

*Moreover, it is not clear how the predictions from uniformization can differ from racing exponentials, other than allowing for a probability to stay in the current genotype.*

The reviewer is correct: the predictions from the time-discretized methods do not differ from their racing exponentials counterparts except in that the former may account for staying in the observed genotype. We now mention this explicitly in the manuscript (section 2.7):

> "Notice that the transition probabilities given by a TD method are proportional to the ones given by its CE counterpart, except for the probability of staying in the current observed genotype which will necessarily be 0 in the CE variants. This difference, however, is critical for some fitness landscapes where fixation can occurr at a fitness maximum (either local or global) that does not correspond to the genotype with all loci mutated. Importantly, the probablities that we are interpreting as corresponding to "staying in the observed genotype" are not arbitrary, but based on the underlying statistical models of the time-discretized methods. These may differ significantly in their predictions for staying in the observed genotype, which can give rise to differences in performance."

*From supplementary section 1.1.1 I gather that staying in the current genotype is in fact a valid answer to "which genotype comes next?". If so, this should be made clear in the main manuscript. I recommend expanding the paragraph in lines 201 - 215 with more detailed explanations.*

The reviewer is correct, and we agree that this point should be clarified in the main text. We have kept the technical details in supplementary section 1.1.1 (so as to avoid compromising the overall readibility of the manuscript in relation to comment #1 by reviewer #2) but added the following clarifications to the main text:

1.1 Section 2.3 of the Methods has been expanded with:

> "Additionally, we allow for a possible answer to the question of "what genotype comes next?" to be "none does". The probability of there not being a "next genotype" is associated with the observation of the final, fixated genotype or, alternatively, with an observed transient genotype that has the same or higher mutational load than the final genotype (see further details in the Supplementary Material, section 1.1)."

1.2 The corresponding passage in section 2.4 of the Methods now reads:

> "We will interpret the diagonal entries of the time-discretized transition matrix as a lower bound on the probability that the ~~given~~observed genotypes behave as local maxima, i.e. that no genotype conforms to our criteria of consecutiveness with respect to the observed one and therefore there is a probability to stay in it."

2. *The considered fitness landscapes seem somewhat limited to draw strong conclusions from. Rough Mount Fuji is essentially a single peak with additional random noise, and representable landscapes are those that correspond to CPMs with directed acyclic graphs (CBN and CAPRI) or trees (OT, CAPRESE). Since the analysis also includes MHN, which is a cyclic graph, I suggest investigating whether one can formulate a corresponding fitness landscape.*

The reviewer is correct that Rough Mount Fuji landscapes are "esentially a single peak with additional noise". However, given that the initial peak can be any genotype, and that the amount of noise can be quite large, the final landscapes can be very different from "a single peak with some noise" and can be strongly multipeaked with many non-accessible genotypes and considerable amounts of reciprocal sign epistasis. This is the case in our fitness landscapes, as can be seen in `https://doi.org/10.1371/journal.pcbi.1007246.s001`, pp. 424 to 634 for seven genes, and pp. 1055 to 1264 for the 10 gene landscapes. And, indeed, it is this flexibility of the RMF model, which allows it to model from very smooth single-peaked landscapes to House of Cards landscapes, that explains its usefulness for modeling empirical fitness ladnscapes [1, 3, 5]. Because of their features (multiple maxima, reciprocal sign epistasis, genotype with all loci mutated not necessarily the fittest one) RMF landscapes cannot be represented by DAGs of restrictions (CBN, CAPRESE, CAPRI), nor by the model of MHN. Given its flexibility, we think it is a fair and reasonable model for the comparison between methods.

The reviewer also indicates *(...) MHN, which is a cyclic graph, I suggest investigating whether one can formulate a corresponding fitness landscape.*.

For models such as CBN, we can formulate representable fitness landscapes as those that have the same accessible genotypes and accessible mutational paths as those implied by the DAG. Because of the way we created these fitness landscapes, setting to 0 the fitness of any non-accessible genotype under the DAG (see section "Fitness landscapes" in [2]), the set of accessible genotypes and paths between genotypes holds even if not under SSWM. For example, even under very large population sizes, no stochastic tunneling is possible because non-accessible genotypes are never created. (It must be noted that there is no reason to expect MHN to perform poorly with these fitness landscapes because, as explained in the original MHN reference [6], MHN can actually perform quite well, or even better than CBN, for models that conform to CBN: see the upper panel of their Figure 4, as well as pp. 2 to 4 of their supplementary material, "Mutual Hazard Networks as stochastic approximations of Conjunctive Bayesian Networks".)

Can we formulate comparable fitness landscapes for MHN? In an SSWM regime, if the MHN is taken at face value as an evolutionary model where its parameters can be interpreted as (functions of) selection coefficients, the implied fitness landscapes are rather unusual.

First, under the MHN model, the probability of transition from any genotype to any other genotype with one additional mutation is always larger than 0 (all $\Theta_{ij}$ are necessarily $> 0$, since $\Theta_{ij} = e^{\theta_{ij}}$). Yes, the probability of such transitions can be very small (for large negative $\theta_{ij}$) but it is, still, larger than 0. As a consequence, under SSWM, the fitness landscape is one where all genotypes are accessible. This would look like a mountain where the peak is located at the genotype with all genes mutated, and any movement (in the sense of increasing mutations) results in a gain in height, even if tiny.

But, in addition, under MHN the fitness of a genotype can depend on the order in which mutations were acquired. This is not just epistasis, since under epistasis the fitness of, say, the genotype with both genes A and B mutated does not depend on whether A or B mutated first. But under the MHN model, unless we restrict the values of $\Theta_{ij}$ so that $\Theta_{ij} = \Theta_{ji}$ whenever $i \neq j$, fitness of a genotype will depend on the order in which mutations were acquired. Why? Let's use the example above. Notice that the path from the WT genotype to AB can be either: $WT \rightarrow A \rightarrow AB$ or $WT \rightarrow B \rightarrow AB$. Along

the first path, the rate of transition of the first transition is $\Theta_{A,A}$ and that of the second is $\Theta_{B,B}\Theta_{B,A}$. Along the second path, however, we have $\Theta_{B,B}$ for the first transition and $\Theta_{A,A}\Theta_{A,B}$ for the second transition. The rates of transition determine the probabilities of transition from one genotype, $i$, to another, $j$, which are a function of the difference in fitness between genotypes $j$ and $i$ [4, 7]. But, as we have just seen, fitness of AB will differ depending on whether one arrives at AB through A or through B.

Put differently, the usual models for fitness combine, often multiplicatively, the contributions of the mutations in each locus and the deviations from the independent contributions. Under one simple parameterizaton, we can write the fitness of genotype AB as, $(1 + s_A)$ $(1 + s_B)(1 + s_{AB})$ (for clarity we have set the fitness of the WT to 1); if $s_{AB} \neq 0$ there would be epistasis (as the fitness of AB would not be just the combination of the independent effects of mutations in each of A and B). But with MHN, the fitness of AB will differ depending on whether we arrive at AB through A or through B, since there is no need for this equality to hold: $\Theta_{A,A}\Theta_{B,B}\Theta_{A,B} = \Theta_{A,A}\Theta_{B,B}\Theta_{B,A}$ because the model does not force $\Theta_{ij} = \Theta_{ji}$ when $i \neq j$.

So fitness landscapes under MHN would be rather unusual fitness landscapes that would not conform to the usual definition of fitness landscapes (a mapping from genotype to fitness): the fitness of any genotype with more than one mutation could possibly differ depending on its actual ancestors. In fact, a genotype with $k$ mutated loci could possibly have $k!$ different fitnesses (one for each of its $k!$ different ways of mutating its $k$ loci).

Note that these issues would not be ameliorated in the absence of SSWM. For example, a fitness landscape that corresponds to MHN should satisfy that, under at least some sensible evolutionary regime, all genotypes are accessible and all transitions from any genotype to any other genotype with one additional mutation are possible, either because the destination genotype has higher fitness, or because the destination genotype is a valley genotype in stochastic tunneling.

Given these features of MHN, we do not think it is intelligible and meaningful to simulate evolution in fitness landscapes that fulfill the MHN model. It is important to emphasize, though, that this does not mean that MHN will work poorly on any other fitness landscape. Rather, we think of MHN as a descriptive, phenomenological model that might actually perform very well in a range of fitness landscapes, but trying to endow the parameters of an MHN model with clear and simple evolutionary interpretations is not possible, in contrast to what happens with, for example, CBN.

Including this long comment in the manuscript itself might not be appropriate, but we have added a new subsection to the Supplementary Material, Types of fitness landscapes considered and their correspondence to the different methods, and we refer to this section from the manuscript:

> "The RMF fitness landscapes usually have multiple local fitness maxima and considerable reciprocal sign epistasis so not even the set of accessible genotypes can be represented by a DAG of restrictions (further details about the fitness landscapes and their correspondence to the methods used are provided in the Supplementary Material, section 1.7)."

## SPECIFIC COMMENTS, MINOR:

3. *Section 2.6 "factors that affect predictive performance" is very difficult to read in prose. I suggest presenting this information as a table.*

   We have included Table 1 and shortened section 2.6 to improve readability.

4. *I don't see the motivation for unweighted versions of CPMs. Since line 348 "For methods with both a weighted and an unweighted version (CBN_uw, MCCBN_uw, OT_uw) using weights never lead to worse performance and sometimes improved it" indicates that they turned out irrelevant, I suggest dropping them from the analysis altogether to make it more assessable.*

   We agree with the reviewer that not showing the unweighted versions will make results more assessable. However, that the unweighted versions perform worse is actually a result of the analysis, not something that we knew with certainty before the study (it is conceivable, for example, that because

of variance-bias trade-offs the unweighted methods might have performed better). So re-analyzing the data after removing the unweighted methods would not be appropriate.

To follow the reviewer's suggestion, what we have done is present in the paper and the supplementary material the figures without the unweighted methods, while leaving in the supplementary material also the figures that include the unweighted methods (so that readers can see, if they wish, that the unweighted methods did not improve performance). Specifically:

4.1 In the results we now indicate:

> "For clarity, these figures do not show methods CBN_uw, MCCBN_uw, OT_uw: for methods with both a weighted and an unweigthed version (CBN_uw, MCCBN_uw, OT_uw) using weights never lead to worse performance and sometimes improved it, as can be seen in the Supplementary material, section 3.4, figures **S4** to **S7**."

4.2 All through the text, we now refer to this new figure in the ms. or the corresponding figures in the supplementary material with the unweighted methods removed (except in the paragraph above, that points to the figures with all methods, so that it can be seen that unweighted methods do not perform better).

4.3 In the supplementary material, section "Linear mixed-effects model trees" we indicate:

> "Two sets of figures are presented. They differ in the methods shown. As in the figure in the main manuscript, in subsubsection "All methods, excluding the unweighted" we do not show the "unweighted" methods (CBN_uw, MCCBN_uw, OT_uw) because for methods with both a weighted and an unweigthed version (CBN_uw, MCCBN_uw, OT_uw) using weights never lead to worse performance and sometimes improved it. Under subsubsection "All methods" we also show the output for all methods, including the unweighted CBN_uw, MCCBN_uw, OT_uw (and, thus, we also show the scenario for the figure of the main manuscript that includes the unweighted methods)."

4.4 The figure legends for the figures in the supplementary material that do not show the unweighted methods say so in the legends.

4.5 We have changed the scripts to generate the figures and updated them in the repository.

5. *In line 117 "To examine the implications of our results for the analyses of cancer data, we have analyzed 25 cancer data sets, described in section Cancer data sets" I suggest adding a short description of the concrete analysis that was performed on the biological data sets.*

We have added the following clarifications to the main text:

5.1 A short description to the Methods overview (section 2.1):

> "To examine the implications of our results for the analyses of cancer data, we have analyzed 25 cancer data sets ~~,~~ by comparing the predictions of different methods and matching them with the patterns seen in the simulated data, as described in section (...)."

5.2 The following paragraph to section 2.7 of the Methods:

> "Because we lack a "standard of truth" in the biological data (i.e. we do not know the true paths of cancer progession, unlike in the simulations, and instead have only a limited number of observations) we aimed to identify specific genotypes from which consecutive tumor progression could potentially be predicted accurately. We studied the similarities across predictions from different methods (measured as the Jensen-Shannon distance of the probability distributions returned by each one) for every feature in the biological data sets. We then compared this similarities with the patterns seen in the simulated data when good performance was possible."

## Reviewer #2

*In this manuscript, Diaz-Colugna and Diaz-Uriarte have examined the conditional predictability of short-term evolution of tumors using variety of Cancer Progression Models. Importantly, they have also quantified the effects of fitness-landscape and genotype-specific variables on the predictive performance using linear mixed-effects model trees. This study on its own is interesting and complements the author's recent lines of research (Referenced in the manuscript as: [24], [25], [26] and [27]). However, the writing can substantially benefit from some major re-organizations, and I have some suggestions that might help further clarify the main points and so potentially enhance the overall quality of the paper.*

1. *My major concern is that the paper seems too technical, and its biological relevance is not well motivated. I strongly recommend the authors to elaborate on the biological significance of their work in the initial paragraphs of the introduction section. How does the predictability of the next genotype can lead to more precise therapeutic choice? Does higher predictability ensure more precise stratification of patients? Can they cite the relevant literature that have addressed these topics to some extent? One potentially inspiring paper that is relevant in this regard and the authors have not cited in the current manuscript is the concept of repeated evolutionary trajectories (Caravagna et al. 2018. Detecting repeated cancer evolution from multi-region tumor sequencing data. Nat. Methods. 2018, 15, 707-714.) in which the relevance of repeatability of cancer evolution in precision medicine is more clearly established.*

   We thank the reviewer for his comments. We agree that the interest for predicting consecutive tumor evolution from a clinical perspective was not sufficiently motivated in the Introduction of our original manuscript. We have included new references and further elaborated on the biological significance of our premise. Specifically:

   1.1 The initial paragraph of the Introduction now reads:

   > "~~Accurately predicting tumor evolution would allow for better prognosis and treatment.~~ Predicting the evolution of a tumor is a critical goal of cancer biology. In general, clinical decisions are guided by cytology and molecular markers used for cancer staging, but these can be unreliable due to sampling problems and the inherent heterogeneity of tumors [Fielding et al. 1992]. The emergence of next-generation sequencing tools has brought promise for a better understanding of the dynamics of cancer evolution [McGranahan and Swanton 2017], and has fueled the development of methods aimed to predict tumor progression [Schill et al. 2020, Gerstung et al. 2009, Gerstung et al. 2011, Montazeri et al. 2016, Szabo and Boucher 2008, Desper et al. 1999, Ramazzotti et al. 2015, Caravagna et al. 2016, Loohuis et al. 2014]. Unfortunately, the stochastic components in key factors that govern ~~tumor progression~~cancer evolution (mutation, genetic drift, or clonal selection [Greaves 2015, Lipinski et al. 2016, Williams et al. 2018], as well as non-genetic variability [Brock et al. 2009] and the tumor microenvironment [Albini and Sporn 2007]) fundamentally limit predictability~~. T~~ –this is also common to other problems [Lassig et al. 2017, Losos 2018] such as the evolution of antibiotic resistance [Toprak et al. 2012] or virulence [Day and Proulx 2004]. ~~where even small increases in predictive power could be of critical importance for the design of treatment strategies [Palmer and Kishony 2013].~~ Nonetheless, repeated tumor progression across patients indicates that prediction must be possible to some extent [Caravagna et al. 2018]. Even small increases in predictive power could be of critical importance for the design of treatment strategies [Palmer et al. 2013]."

   1.2 Lines 53-56 of the original Introduction have been expanded into a new paragraph further elaborating on the relevance of our question for adaptive therapy and precision medicine:

   > "In fact, conditional predictions on the observed genotype are arguably much more relevant than complete evolutionary path predictions for both ~~adaptive therapy [e.g., Melnikov et al. 2020, Hansen et al. 2020, Stankova et al. 2019] and for SMART (sequential, multiple assignment, randomized trial) designs in dynamic treatment regimes~~

for personalized medicine [Tsiatis et al. 2020, Chakraborty et al. 2013]SMART (sequential, multiple assignment, randomized trial) designs in dynamic treatment regimes for personalized medicine [Tsiatis et al. 2020, Chakraborty et al. 2013], and for adaptive therapy [e.g., Melnikov et al. 2020, Hansen et al. 2020, Stankova et al. 2019]. Most cancer treatments aim for an immediate response that maximizes the killing of diseased cells. However, it has been pointed that this approach may favor the proliferation of resistant clones by removing competition, raising the need for alternative, more targeted therapeutic strategies [Gatenby 2009, Enriquez-Navas et al. 2015, Hansen and Read 2020]. For example, intermittent androgen deprivation cycles have been suggested to prevent or delay the expansion of resistant mutants in prostate cancer [Hussain et al. 2013]. Furthermore, multiple studies have aimed to "steer" the evolution of tumors towards states of increased treatment susceptibility, for instance through the sequential application of immunotherapy and chemotherapy in small cell lung cancer [Antonia et al. 2006]. Even when this "steering" is not possible, predicting the short-term evolution of a tumor could allow physicians to anticipate treatments aimed to suppress the proliferation of specific, more aggresive clones predicted to evolve consecutively [Stankova et al. 2019]. Short-term predictions could also aid in patient stratification for personalized medicine, e.g., a clinical study has suggested that forecasting subsequent mutations in precursor gliomas could serve to classify tumors into later-developing glioblastoma subgroups [Ozawa et al. 2014]. "

The paper by Caravagna et al. that the reviewer mentions is indeed very relevant for our work –we have additionally referenced it in the Results section, as detailed in the response to comment #5 of this reviewer.

2. *The manuscript will certainly benefit from some major re-organizations, especially in the results and discussion sections. I recommend the authors to move their real data analysis from the discussion section (lines 445-489 including figures 3 and 4) to the results section and divide the results section into at least two subsections: 1. Simulation results, 2. Real data analysis. In this way, the authors will have better opportunity to provide forward-looking perspective on the biological relevance of their findings in the discussion section, which is currently missing to some extent.*

We agree with the reviewer that the previous organiaztion of the paper was confusing and made the discussion rather dense. We have moved the analysis of the cancer data sets to a new section within the Results.

We have also expanded our Discussion to further elaborate on our work's forward-looking perspectives and how it may be related to the concept of repeated evolutionary trajectories. The response to comment #5 of this reviewer contains a detailed description of the changes introduced.

3. *I think the phrase "Best achievable performance" in line 297 (page 6, the last paragraph) is quite vague and the reader needs to be reminded what exactly it means. I suspect that it is defined in lines 236-237 at page 5: "In our models, the dependent variable is the minimal JS over all methods". Similarly, I think including this definition in the caption of figure 2 will be beneficial, because I guess the JS values in the box plots are minimized across all methods.*

We have followed the reviewer's suggestions: we have clarified the meaning of "best achievable" (and we now use "best possible" consistently through the text, instead of using both "best possible" and "best achievable") and we have fixed the figure legend to emphasize the meaning of the dependent variable and its interpretation. Specifically:

3.1 In what were formerly lines 236-237, we now use, for the first time, the idea of best achievable performance.

> "In our models, the dependent variable is the minimal $JS$ over all methods (for each genotype by replicate by id by detection regime by sample size); this constitutes, therefore, the best possible performance over the set of all methods considered."

3.2 Lines formerly starting at line 297 have be rewritten as

> "~~Best achievable performance is generally larger (i.e., $JS$ is lower)~~The minimal $JS$ over all methods considered (i.e., the best possible performance) generally improves in the representable fitness landscapes compared to ~~than in~~ the local maxima and RMF fitness landscapes (...)."

3.3 We have changed the legend of Fig. 2 as:

> "In the fitted models, the dependent variable is the minimal $JS$ over all methods considered (i.e., the best possible performance): values closer to 0 represent better performance. Internal nodes show the partitioning variables and the values that lead to each partition; light blue denotes the values or categories that lead down the left split and salmon the values or categories that lead down the right split (partitioning criteria are also shown in the edges leading down a node). Leaves or terminal nodes show (weighted) boxplots of the dependent variable, the minimal $JS$ over all methods considered, (...)"

and have made an identical change in the figures in the supplementary material.

3.4 In the rest of the manuscript, we have also replaced all instances of "achievable" by "possible", so as to keep the wording consistent with that used in the introduction, were we write "for this question, the key response variable is the best possible performance over all CPMs considered."

We think that with these changes it is now unambiguous that the dependent variable in the models, the minimal $JS$, is the best possible (or achievable) performance, and this is the variable which is the key focus of the paper.

4. *In figure 2, especially in nodes 10 and 12, we can observe that the average $JS$ score for most of the individual methods is almost one. Would the authors comment on how this is possible? To my view, this might either be a technical error or more likely an interesting observation that deserves to be further elaborated and discussed.*

   It is the second case. We now explain it in the ms.:

   > "The extremely poor performance of the non-TD methods in nodes 10, 12, and 13 in Fig. 2 is in fact explained by the very large frequency of local maxima. Nodes 10, 12, and 13 correspond to genotypes with five or fewer mutations (all descend from the left split of node 1). The genotypes in node 10 are all local maxima (fitness rank of 1, with five or fewer loci mutated). Nodes 12 and 13 correspond to genotypes that are very often ($> 0.5$) or almost always ($> 0.885$) local maxima. For all cases in node 10, for virtually all cases in node 13, and for most of the cases in node 12, only the TD methods can do an acceptable job (predict local maxima) whereas the non-TD methods necessarily will predict a transition to another genotype, leading to $JS$ values of 1."

5. *Obviously because of the lack of standard of truth, the authors have limited their real data analysis on comparing pairs of methods for calculating the JS scores. However, one way to overcome this limitation is to choose a proxy that can reflect the true transition probabilities. For example, the concept of repeated evolutionary trajectories that I mentioned above can be greatly helpful here (Caravagna et al. 2018. Detecting repeated cancer evolution from multi-region tumor sequencing data. Nat. Methods. 2018, 15, 707-714.). Are the evolutionary steps with higher transition probabilities overlap with the repeated evolutionary trajectories reported in the above study? Which of the 13 methods can retrieve a higher fraction of the repeated evolutionary trajectories?*

   *Alternatively, the authors can further dig into the set of interesting genotypes that they have identified and described in lines 473-475: "This same pattern —predictions of methods within the same group are similar and predictions of methods from different groups are not— is observed in the biological*

*data for a small subset of genotypes.". Is it possible for the authors to catalogue those genotypes for each cancer type? Is it possible to detect some biologically interpretable patterns from those genotype sets? Can those genotypes be somehow connected to the concept of repeated evolutionary trajectories? Is there a chance to utilize them for precise stratification of patients and predict the outcome of therapies?*

The reviewer raises a very good point. In fact, in our work we calculate the true transition probabilities in the simulations by aggregating multiple runs of a same evolutionary process as we explain in the Methods. Both our approach and that of Caravagna et al. rely on the same underlying idea that "tumors in different patients can represent multiple instances of the same evolutionary process" [Caravagna et al. 2018]. Therefore, we do expect that the transition probabilities as they are defined in our work map to the repeatability of evolutionary steps sensu Caravagna et al.

It is, however, difficult to perform this mapping in practice. Inferring repeated evolutionary steps requires sequencing data from multiple regions of each tumor. Of course, data from different regions are not independent because they correspond to a same instance of the evolutionary process (and this is in fact what Caravagna et al. exploit in their work). From our perspective, CPMs work under the assumption that the samples provided to them are the result of sequencing $n$ independent tumors. How do we reconcile CPMs requiring cross-sectional sequencing data and repeatability inference requiring multi-region sequencing data? How much worse (if at all) do CPMs perform when the $n$ samples are not independent? It may make sense to subsample so that only one sample per tumor is provided to the CPMs. This, however, will inevitably reduce the sample size ($n$ = number of patients, instead of $n$ = number of patients × number of samples per patient) which has been shown to generically hamper the performance of CPMs [Diaz-Uriarte & Vasallo 2019, and the work reported here]. Another alternative would be to perform several rounds of subsampling and somehow aggregate the outputs of the CPMs across all rounds. Would this make up for the decrease in performance due to the reduced $n$ in each individual round?

These questions are not trivial and must be carefully considered (maybe with the aid of simulated data as well) before assessing the overlap between CPM predictions and repeatable evolutionary steps. However, we do not think that addressing them here would benefit our manuscript. As the reviewer pointed out, this is an already dense and technical paper. Tackling these questions would require elaborating on an additional set of technical concepts and quite possibly compromise readability. We do think that the reviewer's suggestion is definitely exciting and intriguing, and might very well be worth of a separate paper. For that reason, we have made the following changes to the manuscript:

5.1 As the reviewer also suggests, we have catalogued the genotypes exhibiting potential for good CPM performance (i.e. those for which predictions of methods within the same group/across different groups are similar/dissimilar) in a new section in the Supplementary Material. Our repository has been updated with the code to extract this catalog of genotypes. The Results section now points at the Supplementary Material section where the catalog is described:

> "This same pattern —predictions of methods within the same group are similar and predictions of methods from different groups are not— is observed in the biological data for a small subset of genotypes— (listed in section 3.7 of the Supplementary Material)."

5.2 We have significantly expanded our Discussion referencing this catalog of genotypes, as well as elaborating on the idea of mapping transition probabilities and repeated evolutionary steps:

> "As is common to simulation work (...). Our approach allowed us to identify specific genotypes in biological cancer data sets that show potential for accurate consecutive prediction. The two data sets with most genotypes exhibiting this potential (note that not all data sets contain any) correspond to colorectal cancer (Col_pa and Colon_CHG, see section 3.7 of the Supplementary Material), perhaps indicating that tumor progression is particularly predictable (at least locally) in these. In all data sets, genotypes exhibiting this pattern generally have a high mutational load (number of mutations between 0.6 and 0.9 times the total number of features in the data set), which could potentially mean they are more likely to behave as local fitness maxima –therefore

the sharp differences in predictions from the TD and the CE methods, since only the former are able to model such local maxima.

Of course, validating these hypotheses would require a more careful analysis of local predictability in real cancer data sets. The lack of a standard of truth limits our ability to assess the performance of these methods in real clinical scenarios. One possible way to overcome this limitation would be to choose a proxy that can reflect the true transition probabilities. This could involve assesing the repeatability of specific steps in the paths of tumor progression from multi-region sequencing data [Caravagna et al. 2018, Mcgranahan & Swanton 2017]. The performance of a CPM could be quantified as the overlap between the transition probabilities it predicts and the observed repeatability of the evolutionary steps corresponding to those transitions. These are possibly fruitful avenues for future research provided that data sets of large enough sample size for both cross-sectional and longitudinal analysis become available, or that the performance of CPMs is assesed when powered with multi-region sequencing data – where samples are not independent as multiple can correspond to a same evolutionary process, i.e. a same tumor (see section 3.7 for details)."

5.3 We further elaborate on this topic in section 3.7 of the Supplementary Material:

"(...) unveiling which method (if any) is providing accurate predictions would require a proxy for the true transition probabilites. It has been shown that the repeatability of evolutionary steps in cancer progression can be assessed from multi-region tumor sequencing data [Caravagna et al. 2018, McGranahan & Swanton 2017]. Mapping this repeatability to the transition probabilities provided by the CPMs is a promising approach, but it remains technically difficult: CPMs are designed to work with cross-sectional data from independent samples (patients), while detection of repeated evolutionary steps has been done with multiple samples per patient. It is not obvious how one would adapt multi-region sequencing data for CPM use. One option may be using only one sample per patient, but this would result in a significant loss of samples and could compromise the performance of CPMs (as we report here and in previous work, sample size is a major driver of CPM performance [Diaz-Uriarte & Vasallo 2019]). Further work is necessary to clarify if and how CPMs can be adapted for multi-region sequencing data, and to confirm whether the transition probabilities in the sense that we have defined them in this work can be mapped to the repeatability of specific evolutionary steps in real tumors."

It would be unwary to extract stronger conclusions from our catalog of genotypes for which accurate prediction is potentially achievable. This is mainly due to three reasons. First, the pattern in the (dis)similarities across method predictions is promising in that it suggests that those genotypes may satisfy the conditions that allow for accurate prediction, but the pattern on itself does not guarantee that this is the case (see Results and Tables **S8** to **S11**). A more detailed examination of the genotypes would be required to determine their properties (e.g. are they local fitness maxima? what is their fitness rank? etc.), and as we discuss in the manuscript we do not currently have the tools to systematically address these questions –even if our work provides a starting point. Second, the number of genotypes that exhibit the pattern is relatively low ($< 20$ genotypes split across 7 data sets) such that the detection of statistically significant biological patterns would be rather nuanced. And third, each data set uses different types of features (nonsynonymous somatic mutations and/or copy number alterations), making it so comparisons across data sets, even if they correspond to the same cancer type, are not straightforward. We therefore find that any strong claims regarding the biological meaning of this catalog would be difficult to justify.

All in all, we think that our discussion on the real cancer data sets has significantly improved thanks to the feedback from the reviewers and from this comment in particular. The section is now better organized, less technical and overall easier to read. Our work is now better contextualized thanks to the improved clarity regarding its biological relevance and forward-looking perspectives.

# References


1. de Visser, J. A. G. M., Krug, J., 2014. Empirical fitness landscapes and the predictability of evolution. *Nat Rev Genet*, **15(7)**:480–490. doi:10.1038/nrg3744. URL http://www.nature.com/nrg/journal/v15/n7/full/nrg3744.html.

2. Diaz-Uriarte, R., Vasallo, C., 2019. Every which way? On predicting tumor evolution using cancer progression models. *PLOS Computational Biology*, **15(8)**:e1007246. doi:10.1371/journal.pcbi.1007246. URL https://journals.plos.org/ploscompbiol/article?id=10.1371/journal.pcbi.1007246.

3. Franke, J., Klözer, A., de Visser, J. A. G. M., Krug, J., 2011. Evolutionary Accessibility of Mutational Pathways. *PLoS Comput Biol*, **7(8)**:e1002134. doi:10.1371/journal.pcbi.1002134. URL http://dx.doi.org/10.1371/journal.pcbi.1002134.

4. Hosseini, S.-R., Diaz-Uriarte, R., Markowetz, F., Beerenwinkel, N., 2019. Estimating the predictability of cancer evolution. *Bioinformatics*, **35(14)**:i389–i397. doi:10.1093/bioinformatics/btz332. URL https://academic.oup.com/bioinformatics/article/35/14/i389/5529151.

5. Neidhart, J., Szendro, I. G., Krug, J., 2014. Adaptation in Tunably Rugged Fitness Landscapes: The Rough Mount Fuji Model. *Genetics*, **198(2)**:699–721. doi:10.1534/genetics.114.167668. URL http://www.genetics.org/content/198/2/699.

6. Schill, R., Solbrig, S., Wettig, T., Spang, R., 2020. Modelling cancer progression using Mutual Hazard Networks. *Bioinformatics*, **36(1)**:241–249. doi:10.1093/bioinformatics/btz513. URL https://academic.oup.com/bioinformatics/article/36/1/241/5524604.

7. Weinreich, D. M., Delaney, N. F., DePristo, M. A., Hartl, D. L., 2006. Darwinian Evolution Can Follow Only Very Few Mutational Paths to Fitter Proteins. *Science*, **312(5770)**:111–114. doi:10.1126/science.1123539. URL http://science.sciencemag.org/content/312/5770/111.
